# The Ogival Palate: A New Risk Marker of Sudden Unexpected Death in Infancy?

**DOI:** 10.3389/fped.2022.809725

**Published:** 2022-04-18

**Authors:** Mathilde Ducloyer, Matthieu Wargny, Charlotte Medo, Pierre-Antoine Gourraud, Renaud Clement, Karine Levieux, Christèle Gras-Le Guen, Pierre Corre, Caroline Rambaud

**Affiliations:** ^1^Department of Forensic Medicine, University Hospital, Nantes, France; ^2^Department of Radiology, University Hospital, Nantes, France; ^3^CHU de Nantes, INSERM CIC 1413, Pôle Hospitalo-Universitaire 11: Santé Publique, Clinique des Données, Nantes, France; ^4^Department of Pediatrics, University Hospital, Nantes, France; ^5^Department of Oral and Maxillo-Facial Surgery, University Hospital, Nantes, France; ^6^Department of Pathology and Forensic Medicine, AP-HP University Hospital Raymond Poincaré, University of Paris-Saclay, Garches, France

**Keywords:** SUDI (sudden unexpected death in infancy), computed tomography, ogival palate, post mortem imaging, obstructive sleep apnea

## Abstract

**Objective:**

Ogival palate (i.e., a narrow and high-arched palate) is usually described in obstructive breath disorder but has been found in infants unexpectedly deceased. We studied the association between ogival palate and sudden unexpected death in infancy (SUDI) on the basis of a computed tomography (CT) evaluation.

**Methods:**

We conducted a monocentric case-control study of children under 2 years of age who died of SUDI, for which a head CT scan and an autopsy were performed between 2011 and 2018. Each case was matched by sex and age (± 30 days) to two controls selected among living children in the same center who benefited from a cranio-encephalic CT scan. Four parameters of the hard palate were measured by CT: height, width, length, and sagittal angle; the height/width ratio was calculated. The presence of an ogival palate was also subjectively evaluated by the radiologists, independently from the measurements. Standardized odds ratios (OR) were calculated using conditional logistic regression models, all expressed for +1 standard deviation (SD).

**Results:**

Thirty-two deceased children were matched to 64 living control children. Mean ages were 5.0 and 5.3 months, respectively. Twenty-eight cases were considered to have died as a result of SIDS. The mean heights of the hard palate were significantly higher in the deceased children [4.1 (± 0.7) millimeters (mm)] than in the living children [3.2 (± 0.6) mm], with OR (+1SD) = 4.30 (95% confidence interval [CI], 2.04–9.06, *P* = 0.0001). The mean widths of the hard palate were 21.0 (± 1.9) mm and 23.2 (± 2.1) mm, respectively, with OR = 0.15 (95% CI, 0.06–0.40, *P* = 0.0001). The mean sagittal angles were significantly more acute in deceased children [134.5° (± 9.3)] than in living children [142.9° (± 8.1)], with OR = 0.28 (95% CI, 0.14–0.56, *P* = 0.0003). The mean height/width ratios were 19.8 (± 3.7) and 14.1 (± 3.3), respectively, with OR = 6.10 (95% CI, 2.50–14.9, *P* = 0.0001). The hard palate was subjectively considered as ogival in 59.4% (19/32) of the cases versus 12.5% (8/64) of the controls.

**Conclusion:**

Radiological features of the ogival palate were strongly associated with SUDI. This observation still needs to be confirmed and the corresponding clinical features must be identified.

## Introduction

Sudden unexpected death in infancy (SUDI) is defined as the unexpected death of a healthy infant under 1 year of age (1). French recommendations extend this definition for children up to 2 years in age (2). This definition includes deaths resulting from a characterized etiology and sudden infant death syndrome (SIDS), which are deaths that remain unexplained after complete post-mortem investigations (i.e., clinical examination, autopsy, imaging, and biological analyses) (3). SUDI is reported to be associated with multiple risk factors, such as age, sex, sleep position, environment, or intercurrent infection (4). The physiopathology of SUDI has been associated with the “triple risk” theory, namely a vulnerable infant in a critical developmental period, confronted with exogenous stress (5). Among these risk factors, the orofacial structure may be involved in the lethal mechanism of SUDI and near-miss unexpected death. A strong association between the ogival palate (i.e., a high and narrow arch palate) and SUDI was first suspected in 2012 (6). Rambaud et al. described a case series of seven children admitted for SUDI, who presented an ogival palate, clinically diagnosed by opening the mouth. An important proportion of these children presented signs of clinical obstructive sleep apnea (OSA) that affected their breathing before death. This first description suggested a strong link between these events and obstructive sleep disorders, such as obstructive sleep apnea (7–9). To date, few studies have analyzed the specific orofacial structure in deceased children (10). At the same time, the development of post-mortem imaging offers new possibilities to explore craniofacial morphology, especially the skeletal disposition of the upper airways (11, 12). The aim of our study is to determine if an ogival palate (i.e., a narrow and high-arch hard palate) is associated with SUDI in a case-control study based on CT evaluation.

## Materials and Methods

### Population

#### Definition of the Cases

In this case-control study, we retrospectively included all children under two years of age with a post-mortem evaluation for SUDI within the Department of Pediatrics and the Department of Pathology at the University Hospital of Nantes, France, between 2011 and 2018. The criteria of inclusion for cases were as follows: children who underwent a head and/or whole-body post-mortem CT scan, with bone and soft-tissue reconstruction and a complete autopsy with histological examination. For each child, we recorded sex, term of birth, age at death, body position at discovery (i.e., prone or supine position), the use of a pacifier during sleep, treatment treated for gastroesophageal reflux, clinical and/or microbiological signs of upper airways infection, and the cause of death (if known).

#### Definition of the Controls

The controls were randomly selected among living children under 2 years of age who benefited from a cranio-encephalic CT scan in the same center between 2017 and 2018. The matching ratio was 1:2 and was based on sex and age (± 30 days). The criteria of exclusion for the control cases were an incomplete exploration of an osseous palate, a major orofacial malformation (i.e., cleft palate or facial dysmorphic features secondary to syndromic diseases), hospitalization for a near-miss unexpected death and/or SUDI before age 2. For each control, we recorded age, sex, the reason for performing the CT scan, and (when available) if the child used a pacifier during sleep.

### Imaging

The hard palate is anatomically defined as the association of the palatine process of the maxilla with the paired palatine bones. No quantitative and/or radiological definition of the ogival palate exists. Four parameters of the orofacial structure were measured for each child using multiplanar and double-oblique multiplanar reconstructions, following the clinical definition of the ogival palate (i.e., a narrow and high-arch palate). The height, angle, and length were measured in a strict sagittal plane along the axis of the vomer. The width of the palate was measured in a coronal plane perpendicular to the hard palate. The measurements were defined as follows:

- Height of the hard palate: the distance between the highest point of the palate and a line connecting the two extremities of the palate.
- Length of the hard palate: the distance between the incisive foramen and the posterior part of the palatine process, following the curve of the palate.
- Angle of the hard palate: the posterior part of the incisive foramen, the higher point of the palate, and the posterior part of the palatine process.
- Width of the hard palate: the distance between lateral edges of the palate, up to the fifth and the sixth dental buds.

**FIGURE 1:**
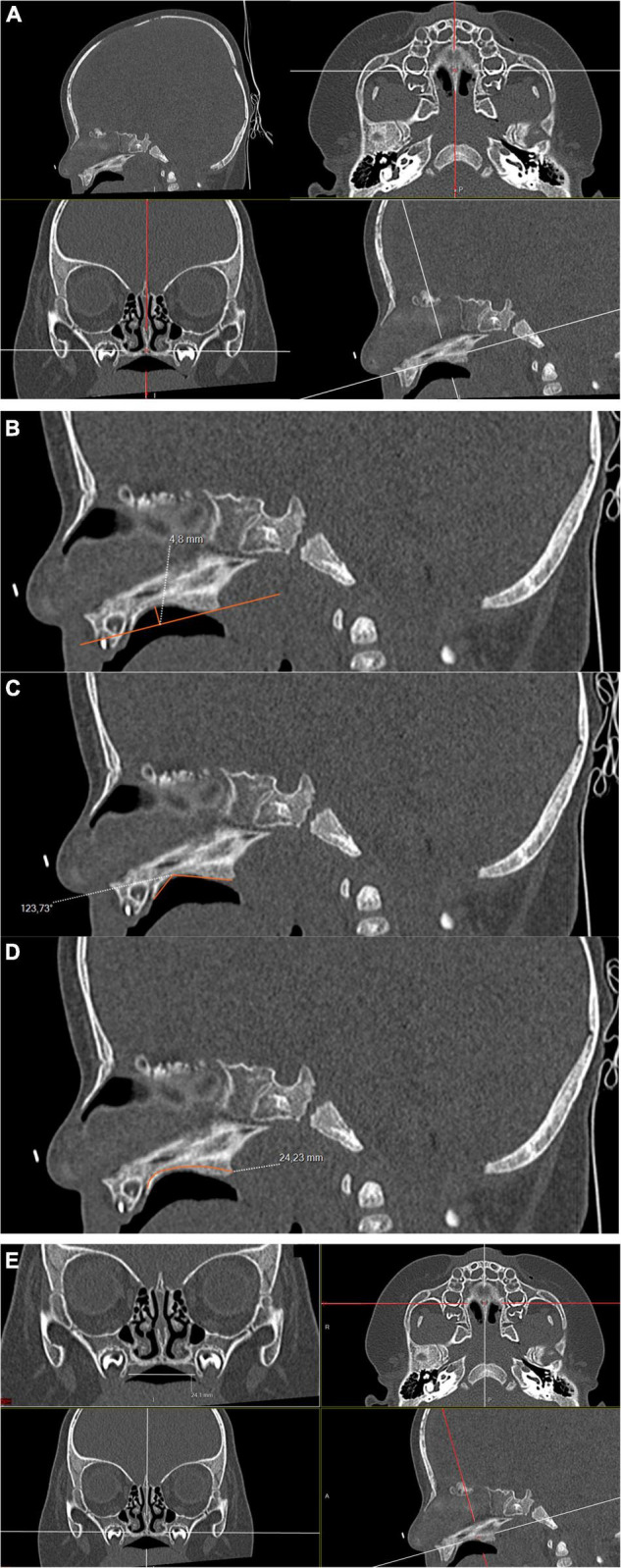
Head CT image of a 5-month-old deceased male with no specific cause of death found after post-mortem investigations; bone filtered, double oblique multiplanar reconstruction; 100 kV, 95 mAs. **(A)** Sagittal plane used to measure the height **(B)**, the sagittal angle **(C)**, and the length of the hard palate **(D)**. **(E)** Coronal plane for the measurement of the width of the palate.

**FIGURE 2:**
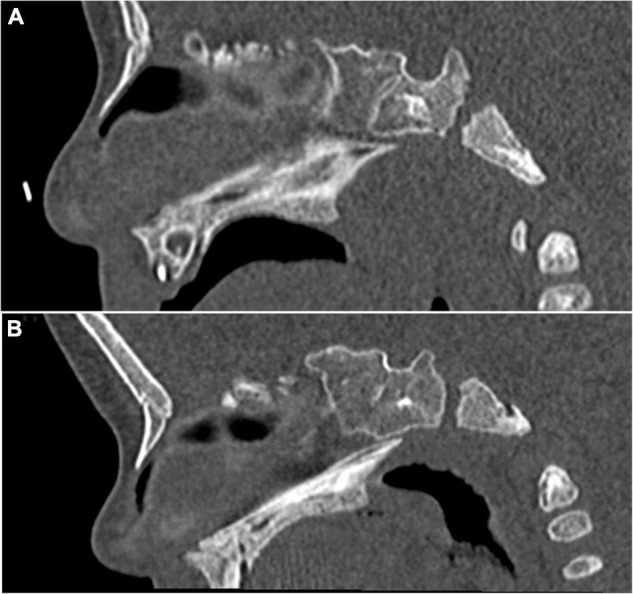
Head CT images for palate comparison. **(A)** Deceased 5-month-old male. Anteroposterior incurvation of the palate can be noticed, with a high palatine arch. The palate was considered to be ogival. **(B)** Living 5-month-old male. CT was performed to eliminate intracranial blood. The palate is almost flat, without anteroposterior incurvation, and was considered to be non-ogival.

We completed this measurement by calculating the height/width ratio (H/W) of the hard palate. We also noted if a uni- or bilateral choanal stenosis was present using an axial plane parallel to the hard palate. The modalities of the measurements are detailed in Figure 1. The median palatine suture, especially the posterior part between the two palatal bones, was evaluated as closed or opened in an axial plane. The presence of an ogival palate was also subjectively evaluated by the radiologist, on the basis of the incurvation of the palate in the sagittal plane and the narrowness between the dental buds in the coronal plane, independently from the measurements. On the opposite, a flat and wide palate was considered normal (Figure 2).

All criteria were evaluated by two independent radiologists, a junior (C.M.) with a year of experience in forensic radiology and a senior (M.D.) with six years of forensic radiology. Each radiologist was blinded to the results of the other.

### Ethical Consideration

Ethical approval for this study protocol was obtained on June 8, 2020, from the Institutional Review Board of the University Hospital of Nantes. Information and written consent from the parents of the deceased children were obtained during hospital admission. Information from the parents of the living children was made through an information booklet delivered during hospital admission. We followed the Strengthening the Reporting of Observational Studies in Epidemiology (STROBE) guidelines to report this study (STROBE checklist).^1^

### Statistical Analyses

Categorical variables are presented using frequencies (%). The quantitative variables are presented using mean (± standard deviation, SD) if the distribution was considered as gaussian or median (25–75th percentiles) in case of a skewed distribution.

All the analyses were based only on the measurements made by the senior radiologist; similar results were obtained from the junior radiologist (data not shown). The inter-rater reliability between the junior and senior radiologist was assessed by intraclass correlation coefficients (ICC, two-way model with random effects, single unit by rater). Scatter plots and Bland-Altman plots are presented for all five parameters of interest.

To assess separately the association of the different study parameters with the group (case or control), we used univariable conditional logistic regression. A multiple conditional logistic regression was also proposed using both the palate’s height and width in the model. All quantitative parameters were standardized (*f*:*x*→(*x*−*mean*(*x*))/*sd*(*x*)), so the associated odds ratios (OR) were expressed for an increase of 1 SD. The associated *P*-values were calculated using a Wald test. These calculations were also completed by analyzing the area under the receiver operating characteristics (AUROC) curves, presented in the Supplementary Material. Considering a global alpha risk set to 5%, the unfavorable hypothesis of 5 uncorrelated tests, and the conservative Bonferroni’s correction, a *P*-value <0.01 (0.05/5) was considered statistically significant. All analyses were performed using the R statistical software version 4.0.0, with the “survival” package (13, 14).

## Results

### Population

The study included 34 children who died between 2011 and 2018 (cases). All the children deceased from SUDI in this period benefited from a head and/or whole-body CT scan. Two of these children were excluded because of an incomplete exploration of the hard palate (*n* = 1) or no bone reconstruction (*n* = 1). The study flowchart can be found in Supplementary Figure 1.

The mean gestational age at birth was 38.8 weeks of amenorrhea (WA), with two children under 37 WA. The position of discovery was available in 27/32 cases (84%): supine position in 4 cases (15%), prone position in 21 cases (78%), and lateral position in 2 cases (7%). Arguments of upper airways infection were found for 11 children (34%). A specific cause of death was identified in 4/32 cases (13%): one hypertrophic cardiopathy, one dilated cardiopathy, one acute gastroenteritis in an ex-premature infant, and one asphyxiating thoracic dystrophy. Two of the children were known to have respiratory disorders while alive. The other 28 cases were considered to have died as a result of SIDS. None of the deceased children had clinical facial dysmorphia. One child was treated for gastroesophageal reflux.

The 32 deceased children were matched with 64 alive children (controls). Among them, two were excluded and replaced, one because of movement artifacts and one because of the child’s death a few days later. They were replaced by two controls using the same matching criteria. CT indications were as follows: suspected intracranial bleeding after accidental or inflicted trauma (*n* = 43, 67%), convulsions (*n* = 8, 12%), subdural enlargement or increased head circumference (*n* = 3, 5%), suspicion of craniostenosis that was refuted by CT imagery (*n* = 3, 5%), infection (meningitis, ethmoids) (*n* = 2, 3%), vomiting (*n* = 2, 3%), hypotonia (*n* = 1, 2%), suspicion of thrombophlebitis (*n* = 1, 2%), and staging of neuroblastoma (*n* = 1, 2%).

The median age of the deceased and living children was 5.0 and 5.3 months, respectively. The average absolute value of the age difference between cases and controls was 0.41 months (0.30), with a maximum difference of 28 days. The male/female ratio was 0.9.

The clinical data of the study population are summarized in Table 1.

**TABLE 1:** Study population data.

Cases (*n* = 32)	
Mean age (minimum-maximum)	5.3 months (2 days – 19 months)
Sex (female)	17/32 (53%)
Mean gestational age at birth (minimum-maximum)	38.8 WA (25–41 WA)
Position of discovery after death	
Prone	21 (65%)
Supine	4 (13%)
Lateral	2 (6%)
Unknown	5 (16%)
Cause of death	
Sudden infant death syndrome	28 (88%)
Hypertrophic cardiopathy	1 (3%)
Dilated cardiopathy	1 (3%)
Acute gastroenteritis	1 (3%)
Asphyxiating thoracic dystrophy	1 (3%)
Controls (*n* = 64)	
Mean age (min-max)	5.0 months (1 day – 19.5 months)
Sex (female)	34/64 (53%)
CT indications	
Suspicion of intracranial bleeding	43 (67%)
Convulsions	8 (13%)
Subdural enlargement or increased head circumference	3 (5%)
Skull deformation	3 (5%)
Infection (meningitis, ethmoïdis)	2 (3%)
Vomiting	2 (3%)
Hypotonia	1 (2%)
Suspicion of thrombophlebitis	1 (2%)
Staging of neuroblastoma	1 (2%)

### Imaging

**FIGURE 3:**
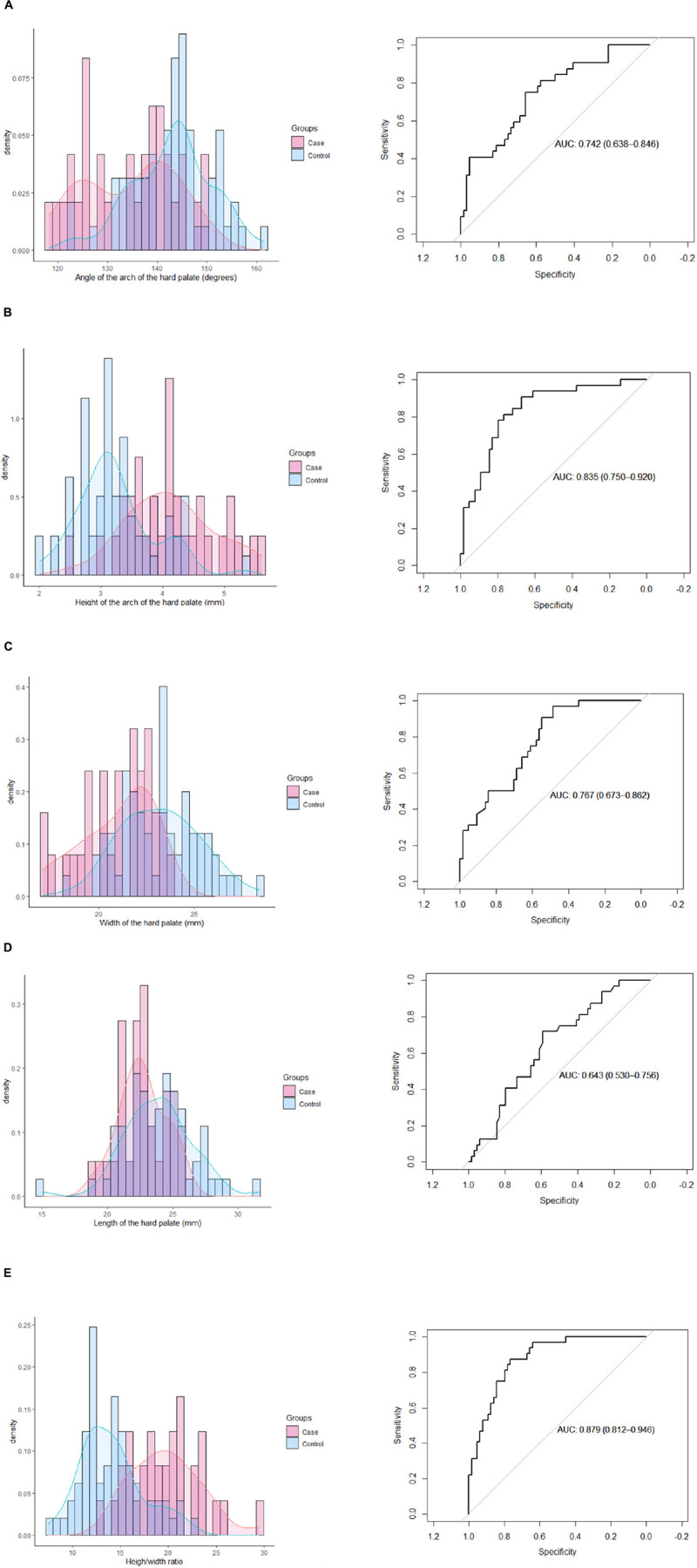
Receiver operating characteristics curves of each parameter. **(A)** Angle of the arch of the hard palate. **(B)** Height of the arch of the hard palate. **(C)** Width of the hard palate. **(D)** Length of the hard palate. **(E)** Height/width ratio.

The mean height of the hard palate was 4.1 millimeters (mm) (± 0.7) in the deceased children versus 3.2 mm (± 0.6) in the living children, with an OR (+1 SD) = 4.30 in univariable analysis (95% CI, 2.04–9.06, *P* = 0.0001) and an OR (+1 SD) = 3.23 after adjustment on the width of the hard palate (95% CI, 1.41–7.37, *P* = 0.0055). The width of the hard palate was 21.0 (± 1.9) mm in the deceased children versus 23.2 (± 2.1) mm in the living children, with an OR (+1 SD) = 0.15 (95% CI, 0.06–0.40, *P* = 0.0001) in univariable analysis and an OR (+1 SD) = 0.18 (95% CI, 0.06–0.60, *P* = 0.0048) after adjustment on the height of the hard palate. The angle of the hard palate was more acute in the deceased children than in the living children, with a mean angle measured at 134.5° (± 9.3) versus 142.9° (± 8.1), respectively, and an OR (+1 SD) = 0.28 (95% CI, 0.14–0.56, *P* = 0.0003). The height/width ratio was higher in the deceased children than in living children at 19.8 (± 3.7) versus 14.1 (± 3.3), respectively, with an OR (+1 SD) = 6.10 (95% CI, 2.50–14.9, *P* < 0.0001). The length of the hard palate was slightly lower in the deceased children (22.7 (± 1.8)) than in the living children (23.9 (± 2.7)), with an OR (+1 SD) = 0.50 (95% CI, 0.29–0.87, *P* = 0.015). These results are detailed in Table 2. The ROC (receiver operating characteristics) curves are presented in Figure 3. The higher AUROC result was found for the height/width ratio, with an AUC = 0.88 (95% CI, 0.81–0.95). The AUC are resumed in Supplementary Table 1.

**TABLE 2:** Factors associated with sudden unexpected death.

	Controls (*n* = 64)	Cases (*n* = 32)	OR (95% CI)	*P*-value
**Univariable analysis**				
Age (months)	5.0 [3.0–7.0]	5.3 [3.0–7.0]	-	-
Female sex	34/64 (53%)	17/32 (53%)	-	-
Hard palate measurements			(for +1 SD)	
Sagittal angle (degrees)	142.9 ± 8.1	134.5 ± 9.3	0.28 (0.14–0.56)	0.0003
Height (mm)	3.2 ± 0.6	4.1 ± 0.7	4.30 (2.04–9.06)	0.0001
Width (mm)	23.2 ± 2.1	21.0 ± 1.9	0.15 (0.06–0.40)	0.0001
Length (mm)	23.9 ± 2.7	22.7 ± 1.8	0.50 (0.29–0.87)	0.015
Height/width ratio	14.1 ± 3.3	19.8 ± 3.7	6.10 (2.50–14.9)	<0.0001
Radiological subjective evaluation of the presence of an ogival palate (narrow, high-arch palate)	8/64 (12.5%)	19/32 (59.4%)	15.1 (3.47–65.7)	0.0003
**Multivariable analysis**				
Height (mm)	3.2 ± 0.6	4.1 ± 0.7	3.23 (1.41–7.37)	0.0055
Width (mm)	23.2 ± 2.1	21.0 ± 1.9	0.18 (0.06–0.60)	0.0048

*CI, confidence interval; mm, millimeters; OR, Odds ratio; SD, standard deviation. Categorical data are expressed using frequencies. (%). Quantitative data are given using mean (± SD) when the distribution was gaussian, except for age (25–75th percentile). Distribution of quantitative variables was assessed on histograms. Only the age was not considered as following a gaussian distribution. The ORs were calculated using a conditional logistic regression accounting of 2:1 matching. For quantitative variables, all ORs are expressed for a +1 SD increase after standardization f:x→(x−mean(x))/sd(x)). P-values were calculated using a Wald test.*

None of the children, living or deceased, had choanal stenosis. The median palatine suture was closed in 9% (3/32) of the deceased children and in 5% (3/64) of the controls; it was considered as opened in all the other children. The hard palate was subjectively considered as ogival by the senior radiologist in 59.4% (19/32) of the cases versus 12.5% (8/64) of the controls. Interobserver reproducibility of this observation was good between the senior and junior radiologists as only 8/96 discordances (8.3%) were noted. The tendency was a slightly higher estimation by the junior radiologist: alive, 15% (9/62); deceased, 63% (20/32). The ICC was 0.68 (95% CI, 0.20–0.85) for the angle, 0.80 (95% CI, 0.70–0.86) for the height, 0.79 (95% CI, 0.70–0.85) for the width, 0.66 (95% CI, 0.35–0.81) for the length, and 0.82 (95% CI, 0.74–0.88) for the height/width ratio.

## Discussion

Our study suggests that a high proportion of the children deceased from SUDI have a particular orofacial anatomy. Following our results, the height of the hard palate was significantly higher and the width significantly lower in the deceased children than in the living children, reinforced by the calculation of the height/width ratio, which seems to be an effective reflection of these anatomical specificities. This quantitative approach was also confirmed by a subjective evaluation of the ogival palate, which showed a significant difference in frequency between the two groups. The high significance of an ogival palate in our results supports the clinical evaluation first made by Rambaud et al. in (6) and is a first step to define it with quantitative measurements. Thus, it strongly reinforces the hypothesis that an ogival palate is associated with SUDI.

Craniofacial modifications are widely studied for their association with OSA (15–17). The optimal functioning of the upper airway (i.e., proper suction and swallowing, as well as nasal breathing) depend on many factors among which normal growth of the facial structures is one of the most important (18). A narrow nasomaxillary complex with an ogival palate can be associated with nasal obstruction and mouth breathing. The misuse of the nasal cavities and mouth breathing may consequently lead to dysfunction of the upper airway muscles, which may exacerbate the abnormalities of craniofacial structure (18–20). An orthodontic correction of this morphotype is one of the effective treatments for OSA, especially in children and young adults (20–22). Thus, if a relation between the orofacial structure and a breathing-related sleep disorder (including OSA and upper airway resistance syndrome) has been established over the years, the physiological process which leads to the morphological variations of the upper airway is debated. Indeed, it remains unclear whether the anatomical changes are the cause or the consequence of the obstructive sleep disorder (23, 24). The palatal morphology is resulting in different factors, which are particularly important during the first months of life (25). Feeding and sucking habits influence the growth of the palate through the forces involved in chewing and swallowing (26). Position and size of the tongue, length of the tongue frenula, strengthens of the masticatory muscles of the mastication are many factors susceptible to impact the palatal development (27, 28). A high vaulted palate has also been described in preterm infants (29). It could be explained by several factors, such as immature swallowing and sucking functions and/or prolonged orotracheal intubation (29, 30). The possibility of the ogival palate as an inherited phenotype must also be considered as a strong hypothesis, as OSA in families has been previously studied (7, 31). Antenatal and neonatal studies could help foster a better understanding of the origins of this anatomical disposition.

The link between OSA and SUDI was first suggested in the 1970s (9, 32–34). The descriptions of the narrowness, obstruction, and increased resistance of the upper airway presented in the literature have suggested an association between OSA and SUDI (31, 35). Dysfunction of the central nervous system has been suspected, too, through alteration of dysautonomic functions, especially in preterm infants; however, this dysfunction is probably less predominant as a lethal process (36, 37). Mechanical asphyxiation that is secondary to an acute upper airway obstruction is indeed recognized as one of the most common mechanisms of death in very young infants, especially suspected when intrathoracic petechiae are numerous (32, 34, 38). The role of airway obstruction is reflected by the effectiveness of prevention campaigns promoting proper sleep environments and sleep position, which mainly aim to reduce possible accidental asphyxiations (39). Thus, it becomes easy to consider that an unfavorable orofacial structure may be a major predisposition for acute airway obstruction when triggered by upper airway infection, by sleeping in a prone position, and/or by using inappropriate bedding (40).

In the current literature, the studies mostly concern school-age children or adults, and the clinical presentation of OSA in very young children is poorly described (41, 42). None of the parents of the deceased children in our study spontaneously described strong signs of breathing disorders during sleep, such as snoring, agitated sleep, or mouth breathing. However, these clinical data may seem banal or insignificant by parents and they are usually not actively asked about during interviews with parents. This information could be more easily gathered by using a standardized questionnaire that asks about OSA symptoms, as detailed by the International Classification of sleep disorders (snoring, obstructed breathing, movement arousals, neck hyperextension during sleep, inward rib-cage motion during inspiration) (43).

This study provides new perspectives to better understand and prevent SUDI. First, studying the orofacial structure in victims of SUDI may help researchers to better understand the protective role of pacifiers or dummies. Many hypotheses have been ventured: pacifiers may help to increase blood pressure during sleep but it is also supposed to enlarge the upper airways thanks to the genioglossus contraction and the mandibular movements (8, 44–46). In addition, the repeated suction and swallowing induced by the use of a pacifier could stimulate the growth and enlargement of nasomaxillary complex and the effectiveness of upper airway muscles (16, 47, 48). The correlation between palate structure and pacifier use in very young children needs to be evaluated in deceased and living children, with long-term cohort studies. Second, abnormalities in oral development could be one explanation for the increased risk of SUDI in preterm infants (49). Preterm children, especially boys, are subject to the alteration of the palatal morphology, which may be increased by lower gestational and longer orotracheal intubation (29). An attentive follow-up of palate structure, suction reflexes, and sleep breathing modality in these children could be an effective way to prevent the development of an ogival palate and may limit the risk of premature death.

Our study presents some limitations. First, the retrospective and monocentric design and the limited size of our sample limit the generalizability of the results, even if the magnitude of the effect allowed for a high statistical significance. A greater number of cases would have facilitated a subgroup analysis, especially regarding the age (less or more than one year). Second, the radiologists were not blinded to the status of the patients. A blind interpretation would have strengthened the results. Third, the choice of a case-control design might be criticized since it implies the comparison of deceased and alive children. However, we believe this design remains the best to investigate our research question with acceptable feasibility. Indeed, the incidence of the SUDI being, fortunately, low, a cohort study would need to recruit tens of thousands of children followed up at least 1 year to identify as much as the 32 presented cases, which would be difficult to implement, and ethically very arguable regarding the associated X-ray exposure. Furthermore, we are confident that the quality of the measurement remains the same, as the death of the child is not expected to modify the assessed radiological parameters. Fourth, the choice of the “controls” remains also an issue. We chose a 1:2 design as it increased the statistical power, and the representativeness of the control population, compared to a 1:1. But ideal controls would have been matched not only for the same age and sex but also with regard to their living environment and other identified risk factors for SUDI such as sleep environment.

The perspectives offered by this preliminary work are numerous. The radiological evaluations of other parameters, such as nasal piriform aperture, nasal septum deviation or mandibular position and measurement, could provide a complete description of the morphotype of children suddenly deceased. Subjective analysis of three-dimensional reconstructions of the hard palate could be very helpful to improve the evaluation of this parameter by different specialists (pediatricians, radiologists, general practitioners). Complementary studies on the correlations between CT images and clinical observations are essential in both living and deceased children to characterize possible subclinical OSA. The frequency of an ogival palate in the normal population of children under 2 years of age, which remains currently unknown, need also be determined, for a more precise interpretation of the frequency of the ogival palate in infants who died unexpectedly.

## Conclusion

The facial structure of the case infants who died of SUDI seems to be different from that of living control children. More specifically, the ogival palate could be the sign of infraclinic OSAs and a narrow structure of the facial upper airways. Thus, the relationship between SIDS and OSA suggests that the orofacial structure could be a supplementary risk factor for SUDI. Recognizing this sign may be the first step in detecting and investigating possible OSAs in infants and children. Further studies are essential to confirm these results, to provide radiological and clinical correlations and to better describe the normal measurements of the nasomaxillary complex in infants. The frequency of ogival palate could consequently be described in the general population of infants. The precise pathogenesis of this anatomical disposition also needs to be determined. At the same time, a more thorough screening for craniofacial growth abnormalities may help to determine their possible involvement in breathing disorders during sleep and avoid premature deaths in very young children.

## Data Availability Statement

The original contributions presented in the study are included in the article/Supplementary Material, further inquiries can be directed to the corresponding author.

## Author Contributions

MD, CM, MW, and CR contributed to the conception and design of the study. PC, CR, and P-AG helped supervise the project. CM and MD organized the database. MW and P-AG performed the statistical analysis and realized some figures. MD and CM wrote the first draft of the manuscript with support from PC and RC. CG-G, MW, and CR wrote sections of the manuscript. All authors contributed to manuscript revision, read, and approved the submitted version.

## Conflict of Interest

The authors declare that the research was conducted in the absence of any commercial or financial relationships that could be construed as a potential conflict of interest.

## Publisher’s Note

All claims expressed in this article are solely those of the authors and do not necessarily represent those of their affiliated organizations, or those of the publisher, the editors and the reviewers. Any product that may be evaluated in this article, or claim that may be made by its manufacturer, is not guaranteed or endorsed by the publisher.

## Footnotes

1
https://www.strobe-statement.org

## Supplementary Material

Supplementary Figure 1 Flow chart detailing the inclusion process and the mean age difference between cases and controls.

Supplementary Table 1 Area under receiver operating characteristic (AUROC) for case identification, results with different hard palate measurements.

## Abbreviations

CT: computed tomography
OR: odds ratio
OSA: obstructive sleep apnea
SUDI: sudden unexpected death in infancy
WA: weeks of amenorrhea.
