# Supplementary figures and images for: The Ogival Palate: A New Risk Marker of Sudden Unexpected Death in Infancy?
Source: Front Pediatr. 2022 Apr 18;10:809725. doi: 10.3389/fped.2022.809725 (PMC9058094; doi:10.3389/fped.2022.809725)

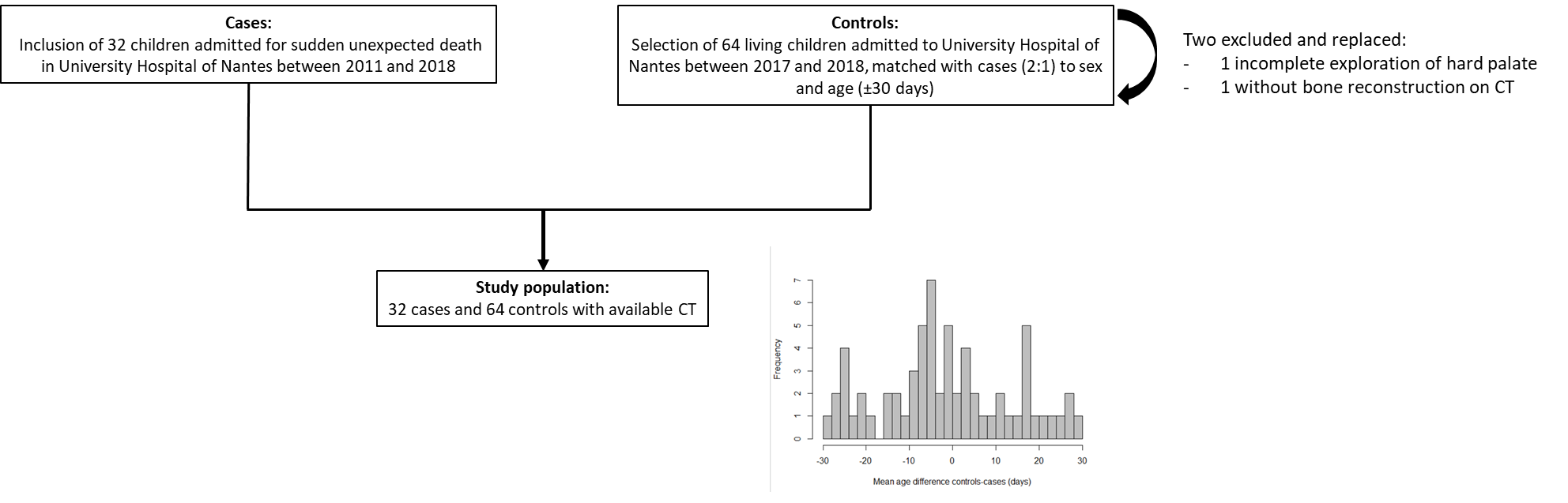

Supplement: Supplementary Figure 1 — Flow chart detailing the inclusion process and the mean age difference between cases and controls. [file Image_1.TIF]
