# Supplementary material for: The Ogival Palate: A New Risk Marker of Sudden Unexpected Death in Infancy?
Source: Front Pediatr. 2022 Apr 18;10:809725. doi: 10.3389/fped.2022.809725 (PMC9058094; doi:10.3389/fped.2022.809725)
Supplement: Supplementary Table 1 — Area under receiver operating characteristic (AUROC) for case identification, results with different hard palate measurements. [file Table_1.DOCX]

**Table 1** (supplemental). Area under receiver operating characteristic (AUROC) for case identification, results with different hard palate measurements

| **Hard palate measurements** | **AUROC** | **95% Confidence Interval** |
| --- | --- | --- |
| Sagittal angle | 0.742 | 0.638-0.846 |
| Height | 0.835 | 0.750-0.920 |
| Width | 0.767 | 0.673-0.862 |
| Length | 0.643 | 0.530-0.756 |
| Height/width ratio | 0.879 | 0.812-0.946 |
